# Improved early risk stratification of deep sternal wound infection risk after coronary artery bypass grafting

**DOI:** 10.1186/s13019-024-02570-9

**Published:** 2024-02-14

**Authors:** Tina Kamensek, Jurij Matija Kalisnik, Mirek Ledwon, Giuseppe Santarpino, Matthias Fittkau, Ferdinand Aurel Vogt, Janez Zibert

**Affiliations:** 1 Faculty of Health Sciences, University of Ljubljana, Zdravstvena pot 5, Ljubljana, 1000 Slovenia; 2 Department of Cardiac Surgery, Klinikum Nuremberg, Paracelsus Medical University, Breslauer Str. 201, 90471 Nuremberg, Germany; 3 Faculty of Medicine, University of Ljubljana, Vrazov trg 2, Ljubljana, 1000 Slovenia; 4 Paracelsus Medical University, Campus Nuremberg, Ernst Nathan Straße 1, 90419 Nuremberg, Germany; 5 Department of Cardiothoracic and Vascular Surgery, University of Graz affiliated Clinic KABEG, Klagenfurt am Wörthersee, Feschnigstrasse 11, Klagenfurt, 9020 Austria

**Keywords:** Coronary artery bypass grafting, Deep sternal wound infection, Risk assessment

## Abstract

**Background:**

Deep sternal wound infection (DSWI) following open heart surgery is associated with excessive morbidity and mortality. Contemporary DSWI risk prediction models aim at identifying high-risk patients with varying complexity and performance characteristics. We aimed to optimize the DSWI risk factor set and to identify additional risk factors for early postoperative detection of patients prone to DSWI.

**Methods:**

Single-centre retrospective analysis of patients with isolated multivessel coronary artery disease undergoing myocardial revascularization at Paracelsus Medical University Nuremberg between 2007 and 2022 was performed to identify risk factors for DSWI. Three data sets were created to examine preoperative, intraoperative, and early postoperative parameters, constituting the “Baseline”, the “Improved Baseline” and the “Extended” models. The “Extended” data set included risk factors that had not been analysed before. Univariable and stepwise forward multiple logistic regression analyses were performed for each respective set of variables.

**Results:**

From 5221 patients, 179 (3.4%) developed DSWI. The “Extended” model performed best, with the area under the curve (AUC) of 0.80, 95%-CI: [0.76, 0.83]. Pleural effusion requiring intervention, postoperative delirium, preoperative hospital stay > 24 h, and the use of fibrin sealant were new independent predictors of DSWI in addition to age, Diabetes Mellitus on insulin, Body Mass Index, peripheral artery disease, mediastinal re-exploration, bilateral internal mammary harvesting, acute kidney injury and blood transfusions.

**Conclusions:**

The “Extended” regression model with the short-term postoperative complications significantly improved DSWI risk discrimination after surgical revascularization. Short preoperative stay, prevention of postoperative delirium, protocols reducing the need for evacuation of effusion and restrictive use of fibrin sealant for sternal closure facilitate DSWI reduction.

**Trial registration:**

The registered retrospective study was registered at the study centre and approved by the Institutional Review Board of Paracelsus Medical University Nuremberg (IRB-2019-005).

**Supplementary Information:**

The online version contains supplementary material available at 10.1186/s13019-024-02570-9.

## Background

Deep sternal wound infection (DSWI) is a life-threatening complication occurring in 1–4% of patients after coronary artery bypass grafting (CABG) [1, 2], associated with lower survival, prolonged hospitalization, higher reoperation rates and resource utilization [2–6]. Prior studies identified risk factors for major infection [6], in particular for DSWI [7–11] to guide decision-making for bilateral internal mammary artery (BIMA) or to identify high-risk patients that would benefit from preoperative intervention strategies for infection reduction [6–16]. Effective prediction of DSWI with external validation was reported in the population undergoing CABG with skeletonized BIMA used in more than 90% [8, 13], but also in a CABG population receiving skeletonized BIMA in less than 20% [9] advancing the use of a predictive scoring system for better preoperative planning. Society of Thoracic Surgeons (STS)-National Cardiac Database study and E-CABG DSWI study developed simplified scoring systems to estimate an individual patient’s preoperative risk for DSWI in general CABG patients with good discriminatory power and comparable c-index of 0.697 and 0.693, respectively [6, 10], providing solid fundament for targeted preoperative preventive strategies. Intraoperative factors were added to develop combined models for improved stratification of major infection risk stratification in general CABG patients and DSWI risk in BIMA patients demonstrating good discrimination with c-index of 0.708 and 0.730, respectively [6, 8]. Nonetheless, less attention has been paid to reassessing DSWI risk after the termination of surgery by the inclusion of early perioperative risk factors or short-term complications. Thus, the present study aimed to develop the most efficient DSWI prediction model by combining pre-, intra- and early postoperative risk factors.

## Methods

### Study population

**Fig. 1:**
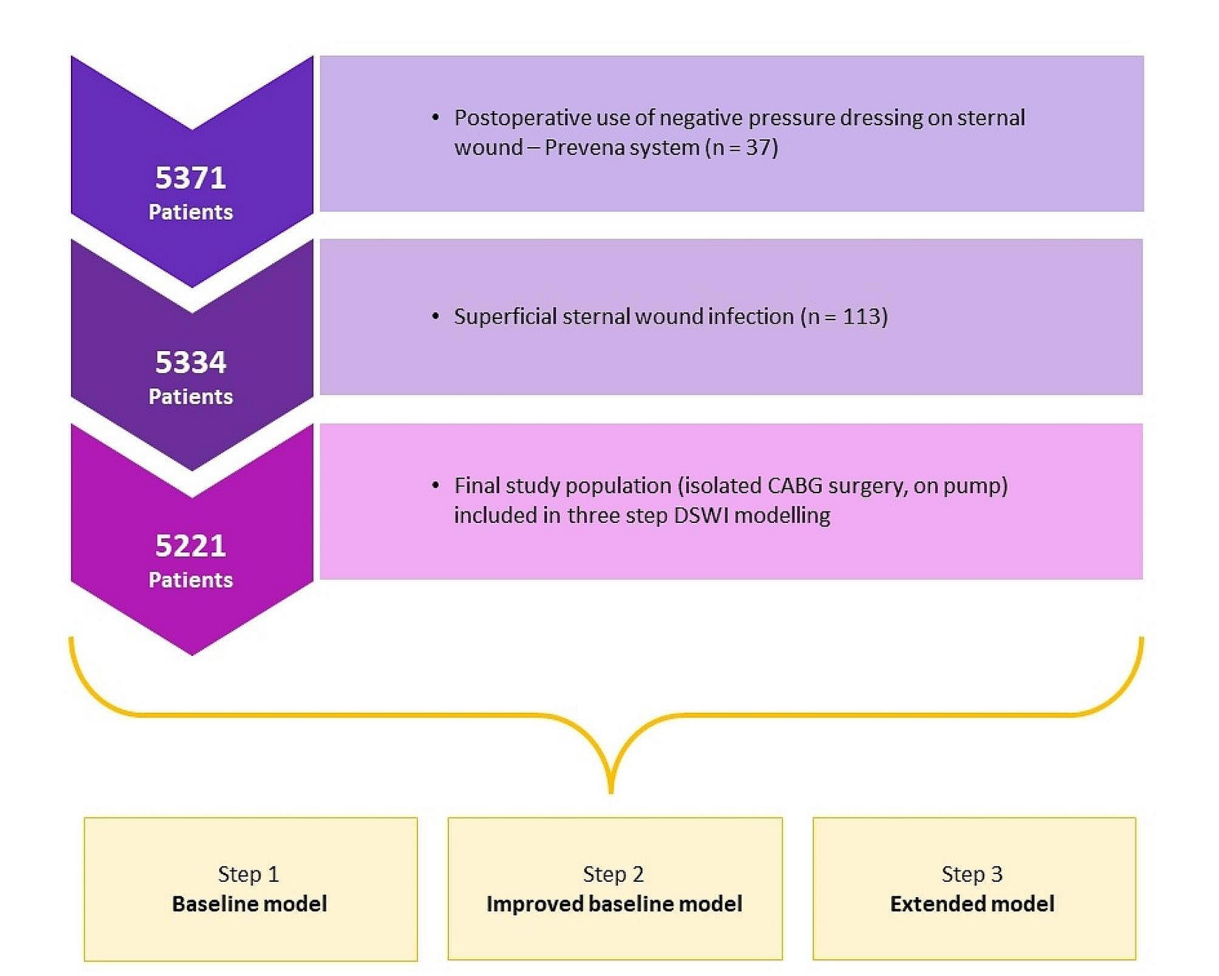
Flowchart of the final study population and exclusion criteria

Between January 2007 and August 2022, 5371 consecutive patients after isolated CABG with cardiopulmonary bypass (CPB) at the Department of Cardiac Surgery at Klinikum Nürnberg, Paracelsus Medical University, Germany, were considered for analysis (Fig. 1). Patients developing superficial sternal wound infection (*n* = 113) or BIMA patients receiving preventive negative pressure dressing (*n* = 37) were excluded from the study (Fig. 1). Pseudo anonymized data of patients were retrieved retrospectively from prospectively managed quality management SAP (Waldorf, Germany) and THG-QIMS database (Terraconnect, Nottuln, Germany). The primary observation was the occurrence of DSWI.

### Construction of variable sets for deep sternal wound infection

Three models were created - the “Baseline”, the “Improved baseline”, and the “Extended” models (Fig. 1). In the “Baseline” model, the variable set from the scoring system of Gatti and colleagues [8] was taken due to its proven externally validated reliability [12, 13, 17, 18]. In the next step, the “Baseline” set was extended by combining risk factors from Gatti and colleagues [8] with risk factors from other scoring systems [7, 9–12] to form the “Improved Baseline” model. In the third step, new variables from our hospital data management system were added to the “Improved baseline” variable set as possible new risk factors, creating the “Extended” model. To minimize the potential impact of collinearity we excluded all categorical variables from the “Extended” set of risk factors derived from scalar variables.

### Definitions

The Centre for Disease Control and Prevention (CDC) classification served to define DSWI, as an infection within 30 days of surgery involving the deep soft tissues (fascial and muscle layers) with or without the involvement of the sternal bone, and organ space infection of the mediastinum, with positive culture results from surgical sites or drains from the mediastinal area or evidence of infection during surgical re-exploration or fever, sternal instability, and positive blood culture results [19].

Unless otherwise stated, the definitions of the included risk factors were adapted from the European System for Cardiac Operative Risk Evaluation II (EuroSCORE II) [20]. Poor preoperative glycemic control was defined as an average basal serum glucose level > 200 mg/dl of available consecutive measurements preoperative. A porcelain aorta was defined as a diffusely calcified and unclampable ascending aorta. Acute kidney injury (AKI) was defined as a serum creatinine increase by ≥ 0,3 mg/dl or by ≥ 26.5 µmol/l from baseline within 48 h after cardiac surgery or an increase in serum creatinine to ≥ 1.5 times baseline within 7 days after cardiac surgery [21]. Low Cardiac Output Syndrome (LCOS) was defined as the pre-, intra- or postoperative decrease of the cardiac index to less than 2.2 L/min/m^2^ requiring inotropic agents and /or mechanical circulatory to maintain the systolic blood pressure higher than 90 mm Hg and the cardiac index greater than 2.2 L/min/m^2^ after optimizing and correcting preload, afterload, electrolyte and blood gas abnormalities [22]. Postoperative delirium was defined as a state in which patients have altered consciousness, orientation, memory, perception, and behaviour [23].

In the “Extended” set of risk factors, we estimated glomerular filtration rate (eGFR) according to the “Modification of Diet in Renal Disease Study” (MDRD) equation [21], rather than the Cockcroft-Gault equation used by Gatti et al. [8] as it more accurately estimates GFR in specific patient populations [24].

All study patients underwent CABG surgery with CPB, therefore “on-pump” was omitted as an independent variable [11]. Multiple blood transfusion (> 2 packed Red Blood Cell-RBCs) was taken instead of “Multiple blood transfusion (of any blood products)” [11].

### Surgery-associated preparations and strategies

Patient preparation consisted of 2% Mupirocin nasal ointment and a 4% chlorhexidine gluconate full antiseptic body wash one day before surgery. Skin preparation was performed with chlorhexidine–alcohol. Surgery was carried out via a median sternotomy with CPB. Multidose cold blood cardioplegia was used for myocardial protection. BIMAs were harvested as semi-skeletonized conduits, whereby the preservation of one of the accompanying mammary veins in respective mammary bed was attempted. Using low-intensity coagulation, conduits were harvested without surrounding muscular tissue, leaving fascia in place from the inferior border of the subclavian vein down to the bifurcation into the superior epigastric and musculophrenic arteries. The BIMA harvesting technique did not change during the study period. BIMAs were used as in situ grafts when possible. Double-loop “figure-of-eight” was preferentially used as a sternal wiring technique. Prophylactic antibiotics were administered before surgery. A first-generation cephalosporin (cefazolin) was usually chosen. Vancomycin was used if there was a severe allergy to β-lactam antibiotics. Bone wax, water-soluble bone wax and fibrin sealants (all Baxter International, Inc., Deerfield, IL, USA) were discouraged and used according to surgeon discretion.

### Management of DSWI

In patients with clinical suspicion of sternal infection, cultures were obtained from the wound sites, sputum, urine, and systemic circulation. Empiric antibiotics were initiated promptly, covering methicillin-resistant Gram-positive, Gram-negative, and anaerobic organisms. The antibiotic regimen was adjusted according to culture results and discontinued by negative culture from the wound after surgical secondary closure and absence of infection, confirmed by normalisation of inflammatory parameters. All patients with diagnosed DSWI received Negative Pressure Wound Therapy (NPWT) plus secondary sternal closure preferentially with re-wiring, sternal plating or muscle flap reconstruction when appropriate.

### Statistical methods

Quantitative variables are reported as mean ± standard deviation or median with interquartile range for non-normally distributed data, and categorical variables are reported as frequencies with percentages. Baseline characteristics were compared between the two cohorts using the χ2 test or Fisher’s exact test for categorical variables and Student’s t-test or Mann–Whitney U-test for continuous variables when normally or non-normally distributed. Normal sample distribution was tested using the Shapiro-Wilk test. A p-value of less than 0.05 was considered statistically significant.

For the “Baseline” set, all variables from the univariable logistic regression analysis were entered into a stepwise-forward multivariable logistic regression analysis. For the “Improved Baseline” and the “Extended” set, significant univariable variables with *p* < 0.1 were fed to a multivariable logistic regression. For optimal model selection, a stepwise approach was used considering at each step one variable for addition or subtraction from the set of explanatory variables in our logistic regression model based on the minimization of the Akaike Information Criteria (AIC) [25]. Thus, the number of explanatory variables in the final model was minimised according to the AIC, while the model retained almost the same accuracy as the model with all variables. In this way, we tried to optimize the number of variables in the models and also remove possible confounding factors in our final multivariable logistic regression models [26]. The odds ratio with a 95% confidence interval (CI) and the numbers of missing values are reported for each variable (see Supplementary Tables 1 and Supplementary Tables 2 - Additional File).

The models were created with “Baseline”, “Improved Baseline” and “Extended” sets of variables, respectively. The multivariable regression models were built by combining preoperative, intraoperative and postoperative data. Evaluations of the models were performed with 10-fold cross-validation by following Transparent Reporting of a multivariable prediction model for Individual Prognosis Or Diagnosis (TRIPOD) recommendations under the Type 2a analysis category with random split sample development and cross-validation [27]. The predictive accuracy of models was assessed by the area under the curve (AUC) in the Receiver Operating Characteristics (ROC) analysis. The models were compared by the method of DeLong et al. [28]. In addition, a forest plot was produced for graphic representation of significant variables with their odds ratios and p-values of the “Extended” model. All statistical analyses were performed using R Statistical Software (version 4.0; R Foundation for Statistical Computing, Vienna, Austria).

## Results

From initially retrieved 5371 patients, 5221 were considered for analysis, with 179 (3.4%) patients developing DSWI. Fifty-four (30.2%) patients with DSWI suffered an associated infection after the primary surgery or at the time of manifest DSWI: 9 (5%) bacteremia/sepsis, 34 (19%) respiratory complication/pneumonia, 6 (3.4%) urinary tract infection and 5 (2.8%) concomitant infection at other sites. From the sternal wound most frequently isolated bacteria were Gram-positive Cocci in 114 patients (47.3%), with Staphylococcus Aureus in 27 (11.2%) and Coagulase-negative Staphylococci in 60 (24.9%) patients (Table 1).

**Table 1:** Microbiological characteristics of the 179 DSWI patients

Microbiology	No. of patients^a^, *n* = 179 (%)
GNB^b^	47 (19.5%)
*Pseudomonas aeruginosa*	2 (0.8%)
*Escherichia coli*	10 (4.1%)
*Enterobacter species*	31 (12.9%)
Other^c^	4 (1.7%)
GPC^b^	114 (47.3%)
*Staphylococcus aureus*	27 (11.2%)
*CNS*	60 (24.9%)
*Enterococcus species*	16 (6.6%)
*Propionibacterium acnes*	4 (1.7%)
Other^c^	7 (2.9%)
Fungi	2 (0.8%)
Polymicrobial	30 (12.4%)
Negative culture	22 (9.2%)
Not determined	26 (10.8%)

CNS = coagulase-negative staphylococcus; GNB = gram-negative bacteria; GPC = gram-positive cocci

^a^ number of patients with etiological diagnosis

^b^ including patients with polymicrobial DSWI.

^c^ GNB: Bacteroides, Haemophilus influenzae, Stenotrophomonas maltophilia;

^c^ GPC: Streptococci, Clostridium difficile, Corynebacterium species,

_Micrococcus luteus

Observed mortality was 2.9% (146 of 5042) in the group without DSWI and 7.3% (13 of 179) in the group with DSWI. Myocardial revascularization was performed using BIMAs +/- saphenous vein in 264 (5.1%) of patients. Supplementary Table 1 (Additional File) represents DSWI risk factors, descriptive statistics and univariate logistic regressions for the entire cohort, included in the “Baseline”, “Improved Baseline” and “Extended” models. Body Mass Index (BMI), Diabetes Mellitus (DM), poor glycaemic control, peripheral arterial disease (PAD), aortic cross-clamp time (min), AKI, prolonged (> 48 h) invasive ventilation, respiratory complications, tracheotomy, postoperative delirium, infection at another site, leukocytes on second postop day (%) and eGFR on second postop day < 60 ml/min, were significant risk factors for DSWI both in the entire cohort.

(see Supplementary Tables 1 and Supplementary Tables 2 - Additional File) as well as in the BIMA subcohort (Table 2).

**Table 2:** Univariate predictors of DSWI in patients with BIMA grafts

Variable	Descriptive statistics				Risk statistics	
	Missing data (n)	No infect (*n* = 243)	DSWI (*n* = 21)	P- value	OR [95% CI]	P- value
BMI	1	27.0 [24.4;30.0]	30.1 [26.9;31.2]	0.027	1.15 [1.03; 1.27]	0.011
Diabetes	2	39 (16.0%)	7 (35.0%)	0.058	2.82 [1.06; 7.51]	0.038
Poor glycaemic control	2	8 (3.31%)	5 (23.8%)	0.002	9.14 [2.68; 31.17]	< 0.001
PAD	1	18 (7.41%)	6 (28.6%)	0.006	5.00 [1.73; 14.45]	0.003
Leukocytes preop (%)	1	7.10 [6,10; 8.30]	8.80 [7.10; 10.5]	0.002	1.44 [1.17; 1.76]	0.001
Infection at another site	1	24 (9.88%)	7 (33.3%)	0.006	4.56 [1.68; 12.41]	0.003
Postoperative delirium	1	12 (4.94%)	5 (23.8%)	0.006	6.02 [1.89; 19.12]	0.002
Number of bypasses	1	3.00 [2.00;3.00]	4.00 [3.00;4.00]	0.001	2.63 [1.45; 4.77]	0.001
Aortic cross-clamp time (min)	1	51.0 [42.0;64.0]	63.0 [58.0;78.0]	0.001	1.04 [1.02; 1.07]	0.001
Acute kidney injury	1	11 (4.53%)	7 (33.3%)	< 0.001	10.55 [3.54; 31.38]	< 0.001
Prolonged (> 48 h) invasive ventilation	1	10 (4.12%)	5 (23.8%)	0.003	7.28 [2.22; 23.86]	0.001
Respiratory complications	1	22 (9.05%)	5 (23.8%)	0.049	3.14 [1.05; 9.39]	0.041
Tracheotomy	6	4 (1.68%)	5 (23.8%)	< 0.001	18.28 [4.47; 74.80]	< 0.001
Leukocytes second postop day (%)	2	10.1 [8.55;12.0]	11.8 [10.1;13.3]	0.015	1.25 [1.08; 1.45]	0.002
eGFR^1^ second postop day < 60 ml/min	1	16 (6.58%)	7 (33.3%)	0.001	7.09 [2.51; 20.06]	< 0.001

Legend: BMI = Body Mass Index; CI = confidence interval; DSWI = deep sternal wound infection; eGFR^1^ = estimated glomerular filtration rate – calculated by MDRD formula; OR = odds ratio; PAD = peripheral arterial disease

From 13 variables, identified as univariate DSWI predictors in a series of Gatti [8], 7 were found significant in our cohort: BMI (kg/m2) > 30, DM on insulin, poor glycaemic control, chronic obstructive pulmonary disease (COPD), congestive heart failure, multiple blood transfusion (> 2 RBCs) and reexploration for bleeding. Further 22 variables from other models were identified as univariate factors for DSWI in our cohort: age, absolute BMI, DM, haemoglobin, anaemia, PAD, left ventricular ejection fraction (LVEF), LVEF < 50%, history of atrial fibrillation (AF), eGFR(ml/min), preoperative hospital stay ˃ 24 h, cardiogenic shock, use of internal mammary artery (IMA), use of BIMA, duration of surgery, aortic cross-clamping time (min), prolonged ventilation (˃ 48 h), respiratory complications, AKI, renal complications, blood transfusion and concomitant infection at another site (Supplementary Table 1, Additional File). Univariate new risk factors for the “Extended” set, excluding categorical derivatives of scalar factors, derived from our patient information management system are presented in Supplementary Table 2 (Additional File). Preoperative infection, use of angiotensin-converting enzyme (ACE) inhibitors, preoperative eGFR under 60 ml/min, postoperative delirium, pericardial drainage, pleural effusion requiring intervention, coagulation disorder, electrical cardioversion for postoperative AF, total drainage (ml), number of received plasma unit > 1, multiple intubation requirement, tracheotomy, leukocyte count on the second postoperative day (%), eGFR on first and on the second postoperative day under 60 ml/min, and use of fibrin sealant were significant univariate factors for DSWI in our cohort (Supplementary Tables 2 - Additional File).

As presented in Tables 3 and 4 of 13 risk factors from the original postoperative Gatti model, DM on insulin, BMI > 30 kg/m^2^, multiple blood transfusions and mediastinal re-exploration came out as independent factors in the “Baseline” model (Table 3 and Supplementary Tables 3 - Additional File).

DM on insulin, BMI (kg/m^2^) > 30, absolute BMI (kg/m^2^), PAD, eGFR, eGFR < 50 ml/min, preoperative hospital stay ˃ 24 h, re-exploration for bleeding, BIMA harvesting, AKI and blood transfusion rounded independent variable set for postoperative “Improved Baseline” model (Table 3 and Supplementary Tables 3 - Additional File).

**Table 3:** Independent predictors of DSWI in patients undergoing Myocardial Revascularisation

Variable	BASELINE MODEL (M1)		IMPROVED BASELINE MODEL (M2)		EXTENDED MODEL (M3)	
	OR [95% CI]	P-value	OR [95% CI]	P-value	OR [95% CI]	P-value
BMI (kg/m^2^) > 30	2.93 [2.12; 4.04]	< 0.001	1.78 [1.04; 3.05]	0.037		
Diabetes - On insulin	2.31 [1.56; 3.42]	< 0.001	1.98 [1.34; 2.92]	0.001	1.88[1.24; 2.57]	0.003
BMI (kg/m^2^)			1.11 [1.05; 1.17]	< 0.001	1.16 [1.11; 1.20]	< 0.001
PAD			1.92 [1.30; 2.83]	0.001	2.19 [1.47; 3.26]	< 0.001
Preoperative hospital stay > 24 h			1.47 [1.04; 2.09]	0.031	1.77 [1.21; 2.57]	0.003
eGFR^1^ (ml/min)			0.99 [0.98; 1.00]	0.033		
eGFR^1^ (ml/min) < 50			0.53 [0.30; 0.93]	0.028		
Age (years)			1.02 [0.99; 1.04]	0.144	1.03 [1.00;1.05]	0.020
Acute kidney injury			1.89 [1.25; 2.85]	0.002	1.89 [1.24; 2.89]	0.003
Multiple blood transfusion (> 2 RBCs)	2.60 [1.76; 3.84]	< 0.001				
Mediastinal re-exploration	3.27 [1.93; 5.55]	< 0.001	3.25 [1.93; 5.48]	< 0.001	3.14 [1.82; 5.41]	< 0.001
Bilateral ITA			5.22 [2.93; 9.31]	< 0.001	4.81 [2.65; 8.73]	< 0.001
Blood transfusion			2.31 [1.60; 3.33]	< 0.001	2.02 [1.38; 2.96]	< 0.001
Fibrin sealant					2.19 [1.10; 4.35]	0.026
Postoperative delirium					2.04 [1.32; 3.16]	0.001
Pleural effusion - intervention					2.26 [1.53; 3.34]	< 0.001

Legend: BMI = Body Mass Index; CI = confidence interval; eGFR^1^ = estimated glomerular filtration rate – calculated by Cockcroft-Gault formula; ITA = internal thoracic artery; OR = odds ratio; PAD = peripheral arterial disease; RBCs = packed red blood cells

Finally, age, DM on insulin, absolute BMI (kg/m^2^), preoperative hospital stay ˃ 24 h, PAD, mediastinal re-exploration, BIMA harvesting, AKI and blood transfusions, application of fibrin sealant for sternal osteoporotic bleeding, postoperative delirium and pleural effusion requiring intervention, rounded the set of 12 independent predictors for “Extended” model (Table 3 and Supplementary Tables 3 - Additional File, Fig. 2).

**Fig. 2:**
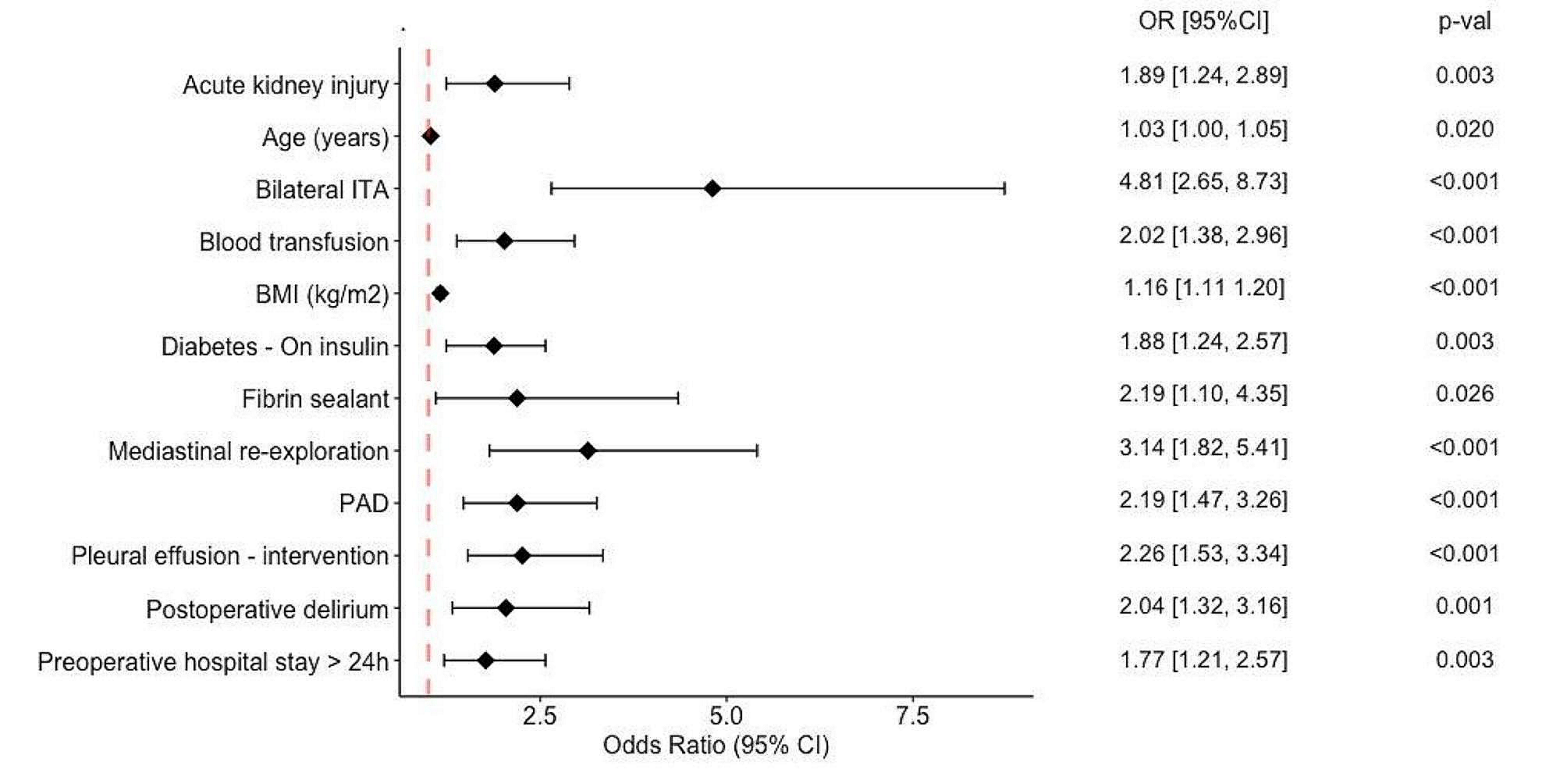
The Forest plot of multivariable analysis of the “Extended” (M3) model

**Fig. 3:**
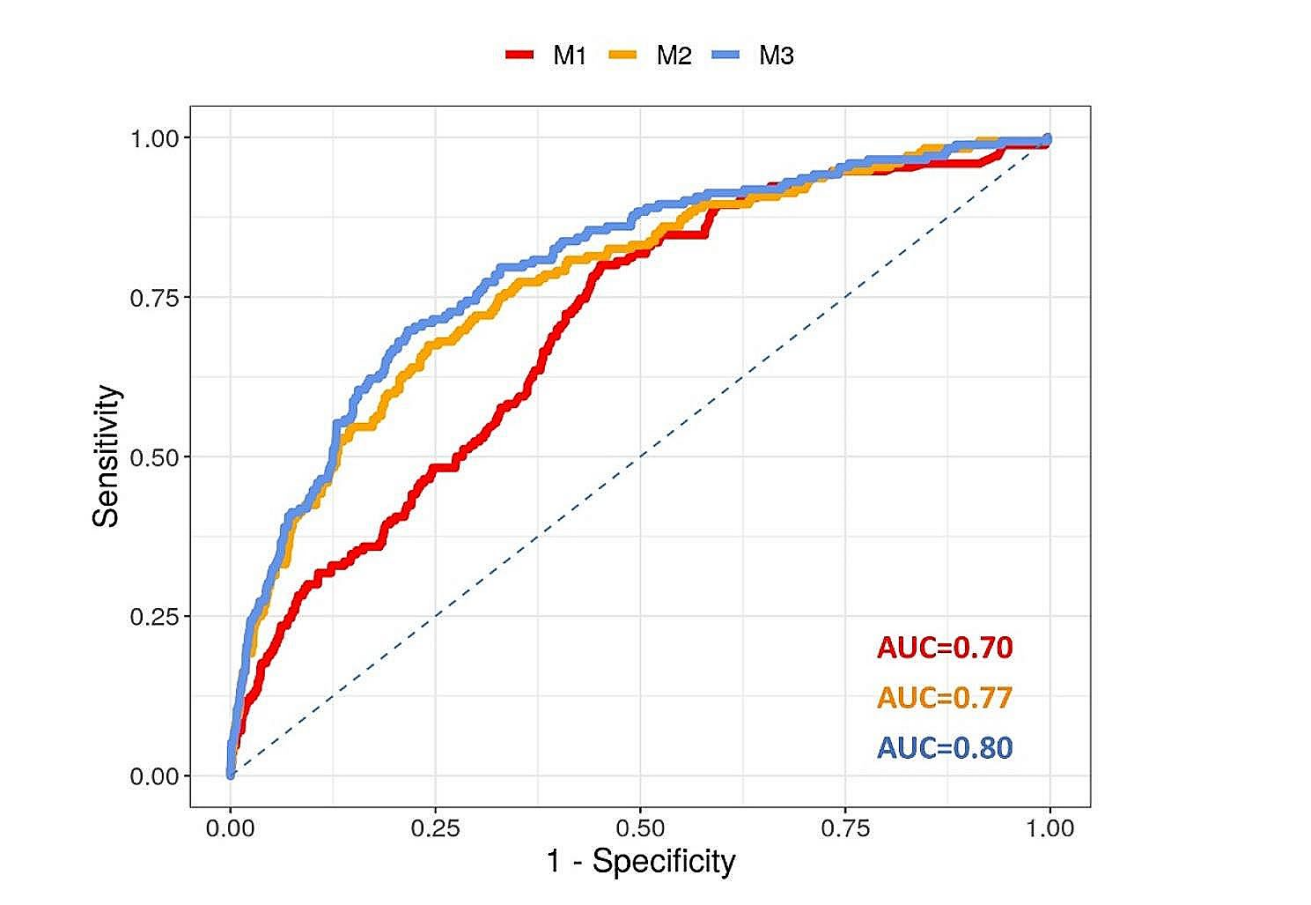
The ROC curves of the “Baseline” (M1), the “Improved Baseline” (M2) and the “Extended” (M3) models using the Test data (AUC = area under the curve)

The AUCs of the “Baseline”, “Improved Baseline” and “Extended” models performed with AUC of 0.70, 95%-CI [0.66, 0.74], 0.77, 95%-CI [0.74, 0.81] and 0.80, 95%-CI [0.76, 0.83], respectively (Fig. 3), the “Extended” model was superior to both the “Baseline” (*p* < 0.001) and the “Improved Baseline” model (*p* = 0.018).

## Discussion

Three main observations can be extrapolated from the current study. First, the application of the Gatti model in the current study (“Baseline”) delivered comparable results to the original cohort [8], supporting its suitability for DSWI risk assessment including subpopulations with lower BIMA utilization. Second, the implemented “Extended” model consisting of 12 independent variables discriminated the patients at risk of DSWI best, in the good-to-excellent range. Finally, pleural effusion requiring intervention and postoperative delirium, as well as the use of fibrin sealant for sternal closure were additional factors, associated with compromised sternal wound healing.

The overall rate of DSWI was 3.4% in our cohort, in a similar range to the reported 4.4% of original the Gatti cohort [8]. Unlike the Gatti cohort, similar proportions of the female gender, chronic dialysis, LCOS and comparable operative risk by EuroSCORE II were observed in the Nuremberg cohort. Despite the existing differences in baseline characteristics and surgical techniques, comparable discrimination power with AUC of 0.70 vs. 0.73 in the “Baseline” model for Nuremberg and the original Gatti cohort, alludes to robustness and potential broader applicability of the model [8]. Externally proven validity with AUC of 0.84 in the French cohort of patients with a 100% rate of BIMA utilization [17] and comparable performance in the Nuremberg cohort with a 5% BIMA utilization rate further supports its clinical application. Concretely, modelling revealed that a BMI value of 30 kg/m^2^ doubled the risk of DSWI (10%), whereby insulin-dependent DM increased the risk of DSWI 3- times to 15%, which has changed our departmental policy strongly discouraging BIMA utilization in these patients.

From 12 independent risk predictors in our final “Extended” model, 5 were associated with comorbidities and preoperative management, 3 directly with surgery and surgical technique and 4 with short-term complications Anticipating that only modifiable risk factors could improve daily routine, the presented study underscores relevancy of considering weight loss programme before elective surgery for the patients with BMI over 30 kg/m2 [3] and maintaining preoperative stays as short as possible.

Leung Wai Sang et all demonstrated that each week of hospital stay preoperatively was associated with a 15% increased risk of mediastinitis [29]. Conklin et al. observed that a preoperative hospital length of stay of more than 7 days was associated with a 4.4-fold increased risk of overall surgical site infections after cardiac surgery [30]. Colonization of antibiotic-resistant nosocomial pathogens, and particularly nutritional deficiency by fasting while awaiting surgery were stated as the most plausible explanations for these observations [29]. Our results demonstrate that already preoperative periods longer than 24 h expose patients to higher DSWI risk.

Similar to other studies [31, 32], our analysis found BIMA harvesting as an independent predictor of DSWI. Major bleeding and sternal wound complications after index surgery occurred more often in the BIMA cohort, which might have neutralised the anticipated benefits of BIMA grafting even longer-term [31], whereby the effects seemed to be more pronounced in obese patients with DM [3, 32]. Given that similar long-term survival benefits of arterial revascularisation might be achieved by using radial artery grafts [31, 33], a proper indication of when to use the right ITA rather than the radial artery seems to be of utmost importance. Of note, our analysis identified independent DSWI predictors AKI, BMI, DM, PAD, and postoperative delirium to be even more potent risk factors for DSWI in the BIMA subcohort. In view of these observations, tight perioperative glucose control and avoidance of BIMA harvesting by concomitant PAD seem to be relevant preventative DSWI measures, whereas particularly BIMA patients could benefit from early detection of AKI and delirium after surgery.

AKI significantly increased the risk of infection including DSWI [34, 35]. Early contemplation of special care bundles reduced the incidence and severity of AKI after cardiac surgery [36]. Renal function preserving protocols could further reduce DSWI, especially when combined with early postoperative detection of AKI [34].

Surgical re-exploration for bleeding and pleural effusions requiring intervention are the two main manifestations of retained blood syndrome, associated with accumulation of fluid around the heart and lungs after cardiac surgery reflecting among other factors inadequate drainage of the thoracic cavity [37–39]. Bleeding complications have been strongly associated with increased transfusion requirements and hemodynamic instability, whereby roughly 70% present with traceable surgical site of bleeding and urgent indication for re-exploration [40]. Conversely, up to 24% of re-explorations are classified as coagulopathic and could be avoided in hemodynamically stable patients [40, 41]. A recent large academic tertiary centre study reported a doubled risk of DSWI in patients after reexploration for bleeding [42]. The strong association of both retained blood syndrome and increased transfusion rates with DSWI in our study supports the premise that standardisation of drainage management including chest tube placement, patency maintenance and timing of removal with improved therapy of coagulopathic diathesis can further reduce DSWI.

Sternal instability “per se” increases the risk of DSWI, whereby external stabilisation reduced the rate of DSWI in a large randomised trial regardless of conventional factors including age, female gender, DM, higher BMI, COPD, renal failure, the logistic EuroSCORE and non-elective indication for surgery [43]. A recent study in high-risk female patients undergoing cardiac surgery [44] demonstrated reduced DSWI when using a postoperative external stabilisation corset [45]. Conversely, intense coughing after surgery alone or in combination with, underestimated pleural effusions are factors promoting sternal instability [46, 47].

The present study not only confirms associations among the aforementioned factors but identifies postoperative delirium as an additional factor. Purportedly, reduced compliance prevents those patients from following instructions and facilitates sternal osteosynthesis destabilization. Recent study findings included in systematic reviews [48, 49] and also American Society for Enhanced Recovery and Perioperative Quality recommendations [50] suggest that early screening for delirium is critical to trigger focused and effective treatment. Non-pharmacological interventions are the first-line management, including reorientation, sleep enhancement, hearing, and vision optimization by using hearing and vision aids, early mobilization, adequate hydration, infection prevention, pain management, and continuous assessment. In contrast, pharmacological options are currently recommended in the second line only and antipsychotics restrictive for hyperactive delirium by individuals who try to harm themselves [49, 50].

Topical hemostatic agents have been suspected to impair postoperative sternal healing possibly promoting sternal instability and even DSWI [51]. Complete bone healing takes up to 6 months after surgery, whereby bone healing was more compromised after the application of bone wax than after the use of water-soluble polymer wax at 3 months after surgery [51, 52] without any increase of sternal infections. Further, the use of fibrin sealants was associated with reduced blood loss in vascular as well as in cardiac surgery, however without significant reduction of hard clinical outcomes such as transfusion, re-exploration for bleeding, or mortality [53, 54]. A recent meta-analysis showed that fibrin sealant application reduced the need for transfusion and blood loss after orthopaedic surgery including total hip or knee arthroplasty without increasing the infection rates [55]. Regarding our study, the application of water-soluble or conventional bone wax has not led to increased DSWI. Conversely, injecting fibrin sealant into the spongy sternum doubled the risk of DSWI.

### Limitations

The present study has inherent limitations due to the retrospective single-institution design. The extended analysis period could have influenced the changes in clinical practice (modification of guidelines for performing surgical techniques, different materials used to perform hemostasis in the dissection of mammary arteries, different antibiotic prophylaxis guidelines and their duration, changes in intensive care treatment, mobilization, length of intensive care unit treatment and length of hospitalization). The authors are aware that all of the above was impossible to take into account in a presented type of study, furthermore, this could influence the results and make the analyzed period not comparable. Further, postoperative complications occur more frequently in complex patients, may be multifactorial and generally reflect worse patients’ conditions, reduced physiological reserves and frailty. Thus, causal explanations of the observed associations are not always possible despite adequately addressing the dilemma in statistical analyses. We addressed the issues of confounding and colinearity by performing a correlation analysis of all our explanatory variables and removing highly correlated variables from the analyses. In addition, all our logistic regression models were built in a stepwise approach to further reduce the effects of collinearity in our factors. Regardless of being collateral, co-modulatory or direct causal agents, taking into account the multifactorial nature of DSWI, primarily modifiable predilecting conditions represented the primary focus of attention in the current study.

Since preoperative serum levels of glycated haemoglobin have not been available for every patient, basal glucose > 200 mg/dl at three preoperative consecutive measurements was adopted as a surrogate marker of poor preoperative glycaemic control. The impact of operative methods such as the off-pump technique on the risk of DSWI could not be evaluated, as patients undergoing myocardial revascularisation using CPB were analysed. The aspects of the bone including osteoporosis, ischemia, the surgeon’s ability, failure to follow the antisepsis procedures, faulty sternotomy and rewiring, and excessive use of an electric scalpel favouring DSWI, were not systematically studied. This study did not evaluate the contribution to DSWI risk of potentially relevant factors such as causative pathogens, antibiotic prophylaxis and preoperative patient preparation. Finally, the study aimed at establishing a model to improve early postoperative identification of patients at risk of any DSWI and as such provides only brief information regarding causative agents and antimicrobial therapy.

## Conclusions

The presented model of 12 independent risk factors complemented with short-term postoperative complications significantly improved DSWI risk discrimination and identified fields of possible clinically meaningful modification. Preoperative hospital stays shorter than 24 h, early detection and first-line nonpharmacological treatment of postoperative delirium, optimised chest tube management reducing retained blood syndrome complications paralleled with restrictive transfusion strategy, application of AKI prevention bundles in high-risk patients, discouraged use of fibrin sealant pave the way to further DSWI reduction.

### Electronic supplementary material

Below is the link to the electronic supplementary material.

Supplementary Table 1: Risk factors for DSWI included in “Baseline ? M1”, “Improved Baseline ? M2” and “Extended ? M3” model.
Supplementary Table 2: Risk factors for DSWI included in “Extended -M3” model.
Supplementary Table 3: Multivariable analysis of all included models.

## Data Availability

The data underlying this article will be shared on reasonable request to the corresponding author.

## Abbreviations

ACE: Angiotensin converting enzyme
AF: Atrial fibrillation
AIC: Akaike Information Criteria
AKI: Acute kidney injury
AUC: The area under the curve
(B)IMA: (Bilateral) internal mammary artery
BMI: Body Mass Index
CABG: Coronary artery bypass grafting
CI: Confidence interval
CDC: Centre for Disease Control and Prevention
COPD: Chronic obstructive pulmonary disease
CPB: Cardiopulmonary bypass
DM: Diabetes mellitus
DSWI: Deep sternal wound infection
eGFR: Estimated glomerular filtration rate
EuroSCORE II: The European System for Cardiac Operative Risk Evaluation II
LCOS: Low Cardiac Output Syndrome
LVEF: Left ventricular ejection fraction
MDRD: Modification of Diet in Renal Disease Study
NPWT: Negative Pressure Wound Therapy
OR: Odds ratio
PAD: Peripheral arterial disease
RBCs: Packed red blood cells
ROC: Receiver Operating Characteristics
STS: Society of Thoracic Surgeons
TRIPOD: Transparent Reporting of a multivariable prediction model for Individual Prognosis or Diagnosis
