# Supplementary material for: Improved early risk stratification of deep sternal wound infection risk after coronary artery bypass grafting
Source: J Cardiothorac Surg. 2024 Feb 14;19:93. doi: 10.1186/s13019-024-02570-9 (PMC10865600; doi:10.1186/s13019-024-02570-9)
Supplement: Supplementary file 1 — Supplementary Table 1: Risk factors for DSWI included in “Baseline ? M1”, “Improved Baseline ? M2” and “Extended ? M3” model. Supplementary Table 2: Risk factors for DSWI included in “Extended -M3” model. Supplementary Table 3: Multivariable analysis of all included models. [file 13019_2024_2570_MOESM1_ESM.docx]

**Supplementary Table 1: Risk factors for DSWI included in “Baseline – M1”, “Improved Baseline – M2” and “Extended – M3” model.**

|  | **Variable** | **M1** | **M2** | **M3** |  | **Descriptive statistics** | | | **Risk statistics** | |
| --- | --- | --- | --- | --- | --- | --- | --- | --- | --- | --- |
|  |  |  |  |  | **Missing data (n)** | **No infect (n=5042)** | **DSWI  (n=179)** | **P- value** | **OR [95% CI]** | **P- value** |
| **PREOPERATIVE** | Age (years) |  | ● | ● | 0 | 69.0 [61.0;75.0] | 70.0 [65.0;76.0] | 0.038 | 1.02 [1.00; 1.03] | 0.049 |
|  | Age (years) >70 |  | ● |  | 0 | 2107 (41.8%) | 85 (47.5%) | 0.150 | 1.26 [0.93; 1.70] | 0.130 |
|  | Female gender | ● | ● | ● | 0 | 967 (19.2%) | 37 (20.7%) | 0.688 | 1.10 [0.76; 1.59] | 0.619 |
|  | Former smoker |  | ● | ● | 14 | 1734 (34.5%) | 61 (34.1%) | 0.974 | 0.98 [0.72; 1.35] | 0.910 |
|  | Current smoker |  | ● | ● | 14 | 1147 (22.8%) | 40 (22.3%) | 0.956 | 0.97 [0.68; 1.39] | 0.884 |
|  | BMI (kg/m^2^) |  | ● | ● | 0 | 27.6 [25.1;30.6] | 30.5 [26.9;34.0] | <0.001 | 1.12 [1.08;1.15] | <0.001 |
|  | BMI (kg/m^2^) <20 |  | ● |  | 0 | 54 (1.07%) | 0 (0.00%) | 0.263 | NA | NA |
|  | BMI (kg/m^2^) >30 | ● | ● |  | 0 | 1425 (28.3%) | 94 (52.5%) | <0.001 | 2.81 [2.08; 3.79] | <0.001 |
|  | Diabetes |  | ● | ● | 30 | 1733 (34.6%) | 92 (51.7%) | <0.001 | 2.03[1.50; 2.73] | <0.001 |
|  | Diabetes - On oral hypoglycaemic agents | ● | ● | ● | 30 | 1134 (22.6%) | 51 (28.7%) | 0.073 | 1.37 [0.99; 1.91] | 0.061 |
|  | Diabetes - On insulin | ● | ● | ● | 30 | 562 (11.2%) | 47 (26.4%) | <0.001 | 2.84 [2.01; 4.01] | <0.001 |
|  | Poor glycaemic control | ● | ● | ● | 48 | 591 (11.8%) | 31 (17.5%) | 0.030 | 1.58 [1.06; 2.35] | 0.023 |
|  | Hypertension |  | ● | ● | 5 | 4772 (94.7%) | 172 (96.6%) | 0.340 | 1.60 [0.70; 3.64] | 0.264 |
|  | Serum haemoglobin (g/dl) |  | ● | ● | 18 | 14.0 [12.9;14.9] | 13.7 [12.2;14.9] | 0.019 | 0.88 [0.81; 0.96] | 0.003 |
|  | Serum haemoglobin (g/dl) <12 |  | ● |  | 18 | 644 (12.8%) | 39 (22.0%) | 0.001 | 1.92 [1.34; 2.77] | <0.001 |
|  | Chronic lung disease ^a^ | ● | ● | ● | 3 | 611 (12.1%) | 32 (17.9%) | 0.029 | 1.58 [1.07; 2.33] | 0.022 |
|  | eGFR (ml/min) |  | ● |  | 2 | 77.0 [60.2;97.5] | 73.0 [56.1;91.4] | 0.024 | 0.99 [0.99; 1.00] | 0.035 |
|  | eGFR (ml/min) 50-85 |  | ● |  | 2 | 2420 (48.0%) | 91 (50.8%) | 0.505 | 1.12 [0.83; 1.51] | 0.458 |
|  | eGFR (ml/min) <50 |  | ● |  | 2 | 638 (12.7%) | 31 (17.3%) | 0.086 | 1.45 [0.97; 2.15] | 0.068 |
|  | NYHA III IV |  | ● | ● | 1 | 2674 (53.0%) | 96 (53.6%) | 0.938 | 1.02 [0.76; 1.38] | 0.877 |
|  | Renal disease |  | ● | ● | 2 | 166 (3.29%) | 8 (4.47%) | 0.516 | 1.37 [0.67; 2.84] | 0.391 |
|  | PAD |  | ● | ● | 9 | 672 (13.4%) | 44 (24.6%) | <0.001 | 2.12 [1.49; 3.00] | <0.001 |
|  | LMS |  | ● | ● | 1 | 1824 (36.2%) | 60 (33.5%) | 0.516 | 0.89 [0.65; 1.22] | 0.466 |
|  | History of atrial fibrillation |  | ● | ● | 4 | 373 (7.4%) | 22 (12.3%) | 0.022 | 1.75 [1.11; 2.77] | 0.016 |
|  | Chronic dialysis | ● | ● | ● | 24 | 106 (2.1%) | 6 (3.41%) | 0.280 | 1.64 [0.71; 3.78] | 0.248 |
|  | Congestive heart failure | ● | ● | ● | 5 | 1465 (29.1%) | 70 (39.1%) | 0.005 | 1.57 [1.15; 2.13] | 0.004 |
|  | Unstable angina |  | ● | ● | 1 | 523 (10.4%) | 20 (11.2%) | 0.826 | 1.09 [0.68; 1.75] | 0.731 |
|  | Recent myocardial infarction |  | ● | ● | 2 | 1926 (38.2%) | 79 (44.1%) | 0.128 | 1.28 [0.95; 1.73] | 0.110 |
|  | Prior PTCA |  | ● | ● | 1 | 1187 (23.5%) | 46 (25.7%) | 0.564 | 1.12 [0.80; 1.58] | 0.506 |
|  | LVEF (%) |  | ● | ● | 5 | 60.0 [50.0;64.0] | 55.0 [45.0;61.0] | 0.006 | 0.98 [0.97; 1.00] | 0.005 |
|  | LVEF (%) <50 |  | ● |  | 5 | 1101 (21.9%) | 54 (30.2%) | 0.011 | 1.54 [1.12; 2.14] | 0.009 |
|  | Preoperative hospital stay >24h |  | ● | ● | 0 | 3122 (61.9%) | 125 (69.8%) | 0.039 | 1.42 [1.03; 1.97] | 0.033 |
|  | Cardiogenic shock |  | ● | ● | 1 | 477 (9.5%) | 30 (16.8%) | 0.002 | 1.93 [1.29; 2.88] | 0.001 |
|  | Previous stroke |  | ● | ● | 6 | 407 (8.1%) | 16 (8.9%) | 0.785 | 1.12 [0.66; 1.88] | 0.680 |
|  | Preoperative IABP |  | ● | ● | 3 | 149 (3.00%) | 1 (0.6%) | 0.097 | 1.18 [1.03; 2.33] | 0.093 |
|  | Previous cardiac operation |  | ● | ● | 0 | 59 (1.2%) | 0 (0.0%) | 0.269 | N.A. | N.A. |
|  | Previous CABG surgery |  | ● | ● | 0 | 52 (1.0%) | 0 (0.0%) | 0.263 | N.A. | N.A. |
|  | Urgent surgical priority | ● | ● | ● | 2 | 1909 (37.9%) | 62 (34.6%) | 0.424 | 0.87 [0.64; 1.19] | 0.380 |
|  | Emergency |  | ● | ● | 1 | 226 (4.5%) | 7 (3.9%) | 0.857 | 0.87 [0.40; 1.87] | 0.716 |
|  | Expected operative risk (by EuroSCORE II) (%) |  | ● | ● | 1 | 3.2 [1.8;6.3] | 4.5 [2.5;8.1] | <0.001 | 1.01 [1.00; 1.03] | 0.134 |
|  | EuroSCORE II (%) >10 |  | ● |  | 1 | 662 (13.1%) | 28 (15.6%) | 0.389 | 1.23 [0.81; 1.85] | 0.331 |
| **INTRAOPERATIVE, POSTOPERATIVE** | Porcelain aorta (by intraop. EAS) | ● | ● | ● | 53 | 1274 (25.5%) | 56 (31.5%) | 0.091 | 1.34 [0.97; 1.85] | 0.076 |
|  | Number of bypasses |  | ● | ● | 0 | 3.0 [2.0;3.0] | 3.0 [2.0;3.0] | 0.076 | 1.20 [0.99; 1.46] | 0.071 |
|  | Use of ITA |  | ● | ● | 1 | 4693 (93.1%) | 155 (86.6%) | 0.001 | 0.48 [0.31; 0.75] | <0.001 |
|  | Bilateral ITA |  | ● | ● | 1 | 243 (4.8%) | 21 (11.7%) | <0.001 | 2.62 [1.64; 4.21] | <0.001 |
|  | Duration of surgery (min) |  | ● | ● | 2 | 177 [151;205] | 183 [162;216] | 0.008 | 1.00 [1.00;1.01] | 0.015 |
|  | Cardiopulmonary bypass time (min) |  | ● | ● | 1 | 75.0 [59.0;92.0] | 78.0 [64.0;90.0] | 0.051 | 1.01 [1.00;1.01] | 0.067 |
|  | Cardiopulmonary bypass time >100 min |  | ● |  | 0 | 784 (15.5%) | 30 (16.8%) | 0.738 | 1.09 [0.73;1.63] | 0.661 |
|  | Aortic cross-clamp time (min) |  | ● | ● | 0 | 43.0 [34.0;53.0] | 45.0 [36.0;54.5] | 0.061 | 1.01 [1.00;1.02] | 0.044 |
|  | complete revascularization^b^ |  | ● | ● | 55 | 4313 (86.5%) | 151 (84.8%) | 0.607 | 0.88 [0.58;1.33] | 0.532 |
|  | Prolonged (>48 h) invasive ventilation |  | ● | ● | 75 | 219 (4.4%) | 29 (16.5%) | <0.001 | 4.28 [2.81;6.52] | <0.001 |
|  | Respiratory complications |  | ● | ● | 24 | 497 (9.9%) | 40 (22.5%) | <0.001 | 2.64 [1.83; 3.80] | <0.001 |
|  | Atrial fibrillation, new onset |  | ● | ● | 1 | 568 (11.3%) | 23 (12.8%) | 0.592 | 1.16 [0.74; 1.81] | 0.512 |
|  | Myocardial infarction |  | ● | ● | 13 | 55 (1.09%) | 1 (0.56%) | 1.000 | 0.51 [0.07;3.69] | 0.503 |
|  | Low cardiac output | ● | ● | ● | 21 | 177 (3.5%) | 10 (5.59%) | 0.211 | 1.62 [0.84; 3.12] | 0.149 |
|  | Acute kidney injury |  | ● | ● | 32 | 531 (10.6%) | 53 (29.8%) | <0.001 | 3.58 [2.56; 4.99] | <0.001 |
|  | Renal complications |  | ● | ● | 21 | 248 (4.9%) | 26 (14.5%) | <0.001 | 3.27 [2.12; 5.05] | <0.001 |
|  | 24-h Chest tube drainage (ml)^a^ |  | ● | ● | 0 | 0.0 [0.0;45] | 0.0 [0.0;45] | 0.673 | 1.00 [1.00; 1.00] | 0.821 |
|  | 24-h Chest tube drainage/weight (ml/kg)^a^ |  | ● | ● | 0 | 0.0 [0.0;5.3] | 0.0 [0.0;4.7] | 0.559 | 0.99 [0.96; 1.03] | 0.689 |
|  | Blood transfusion |  | ● | ● | 0 | 1930 (38.3%) | 115 (64.2%) | <0.001 | 2.90 [2.12; 3.95] | <0.001 |
|  | Multiple blood transfusion (>2 RBCs) | ● | ● | ● | 0 | 644 (12.8%) | 56 (31.3%) | <0.001 | 3.11 [2.24; 4.31] | <0.001 |
|  | Infection at another site |  | ● | ● | 0 | 640 (12.7%) | 38 (21.2%) | 0.001 | 1.85 [1.28; 2.68] | 0.001 |
|  | Mediastinal re-exploration | ● | ● | ● | 39 | 174 (3.5%) | 24 (13.6%) | <0.001 | 4.36 [2.76; 6.87] | <0.001 |
| Legend: BMI = Body Mass Index; CABG = coronary artery bypass graft; CI = confidence interval; DSWI = deep sternal wound infection; EAS = epiaortic ultrasonography scan; eGFR = estimated glomerular filtration rate; IABP = intra-aortic balloon pump; ITA = internal thoracic artery; LMS = left main stenosis; LVEF = left ventricular ejection fraction; N.A. = logistic regression was not calculated, due to all cases of individual group were just in one category; NYHA = New York Heart Association Functional Classification; OR = odds ratio; PTCA = percutaneous transluminal coronary angioplasty; PAD = peripheral arerial disease; RBCs = packed red blood cells.  ^a^ Gatti et al. [1] reported data for 48-h.  ^b^ Raja & Benedeto[2] reported data for incomplete revascularization. | | | | | | | | | | |

**Supplementary Table 2: Risk factors for DSWI included in “Extended -M3” model.**

|  | **Variable** | **M3** |  | **Descriptive statistics** | | | **Risk statistics** | |
| --- | --- | --- | --- | --- | --- | --- | --- | --- |
|  |  |  | **Missing data (n)** | **No infect  (n = 5042)** | **DSWI  (n = 179)** | **P- value** | **OR [95% CI]** | **P- value** |
| **PREOPERATIVE**  **PREOPERATIVE** | Hyperlipidemia | ● | 9 | 4538 (90.2%) | 163 (91.1%) | 0.788 | 1.11 [0.66; 1.87] | 0.692 |
|  | Preoperative infection | ● | 2 | 92 (1.8%) | 7 (3.9%) | 0.082 | 2.20 [1.01; 4.82] | 0.048 |
|  | Liver disease | ● | 17 | 73 (1.5%) | 0 (0.0%) | 0.181 | NA | NA |
|  | Mehanic ventilation preop | ● | 2 | 48 (1.0%) | 1 (0.6%) | 1.000 | 0.58 [0.08; 4.26] | 0.596 |
|  | ACE inhibitors | ● | 11 | 2777 (55.2%) | 116 (64.8%) | 0.014 | 1.49 [1.09; 2.04] | 0.012 |
|  | AT II Antagonists | ● | 13 | 847 (16.8%) | 26 (14.5%) | 0.475 | 0.84 [0.55; 1.28] | 0.415 |
|  | Betablocker | ● | 11 | 3523 (70.0%) | 129 (72.1%) | 0.615 | 1.10 [0.79; 1.54] | 0.558 |
|  | Ca Antagonists | ● | 11 | 1094 (21.7%) | 42 (23.5%) | 0.649 | 1.10 [0.78; 1.57] | 0.584 |
|  | Leukocytes preop (%) | ● | 18 | 7.2 [6.1;8.6] | 7.8 [6.5;9.0] | 0.004 | 1.01 [1.00; 1.02] | 0.132 |
|  | Platelets preop (10^9^/L) | ● | 22 | 223 [188;265] | 232 [192;274] | 0.186 | 1.00 [1.00; 1.00] | 0.243 |
|  | CRP preop (mg/L) >3 mg/L^a^ | ● | 97 | 339 (6.8%) | 15 (8.8%) | 0.410 | 1.31 [0.76; 2.25] | 0.330 |
|  | eGFR preop <60 ml/min ^b^ | ● | 23 | 1160 (23.1%) | 66 (37.5%) | <0.001 | 2.00 [1.46; 2.73] | <0.001 |
| **INTRAOPERATIVE, POSTOPERATIVE** | Postoperative delirium | ● | 9 | 374 (7.4%) | 42 (23.5%) | <0.001 | 3.82 [2.66; 5.48] | <0.001 |
|  | Pericardial effusion (ml) | ● | 0 | 0.0 [0.0;0.0] | 0.0 [0.0;0.0] | 0.103 | 0.99 [0.98; 1.00] | 0.261 |
|  | Pericardial drainage | ● | 42 | 1420 (28.4%) | 65 (36.3%) | 0.027 | 1.44 [1.05; 1.96] | 0.022 |
|  | Pleural effusion - intervention | ● | 29 | 643 (12.8%) | 57 (31.8%) | <0.001 | 3.18 [2.29; 4.40] | <0.001 |
|  | Pneumothorax | ● | 35 | 184 (3.7%) | 3 (1.7%) | 0.228 | 0.45 [0.14; 1.41] | 0.170 |
|  | Coagulation disorder | ● | 20 | 119 (2.4%) | 10 (5.6%) | 0.013 | 2.44 [1.26; 4.73] | 0.008 |
|  | Reoperation for bleeding | ● | 22 | 181 (3.6%) | 10 (5.6%) | 0.230 | 1.59 [0.83; 3.06] | 0.164 |
|  | New operation (on pump) | ● | 26 | 23 (0.5%) | 2 (1.1%) | 0.212 | 2.45 [0.57; 10.49] | 0.226 |
|  | Cardioversion | ● | 34 | 180 (3.6%) | 17 (9.5%) | <0.001 | 2.82 [1.67; 4.74] | <0.001 |
|  | Stroke | ● | 24 | 39 (0.8%) | 1 (0.6%) | 1.000 | 0.72 [0.10; 5.25] | 0.743 |
|  | Total drainage (ml) | ● | 0 | 500 [350;700] | 590 [400;800] | 0.005 | 1.00 [1.00; 1.00] | 0.007 |
|  | Number of plasma units >1 | ● | 0 | 302 (6.0%) | 21 (11.7%) | 0.001 | 1.07 [1.02; 1.11] | 0.002 |
|  | Platelet unit number >1 | ● | 0 | 118 (2.3%) | 8 (4.5%) | 0.008 | 1.05 [0.96; 1.16] | 0.294 |
|  | Reintubation | ● | 54 | 138 (2.8%) | 21 (11.7%) | <0.001 | 4.67 [2.87; 7.59] | <0.001 |
|  | Tracheotomy | ● | 107 | 152 (3.1%) | 24 (13.5%) | <0.001 | 4.91 [3.10; 7.77] | <0.001 |
|  | Minimum patient body temperature | ● | 0 | 34.7 [34.1;35.0] | 34.8 [34.3;35.2] | 0.041 | 1.06 [0.91; 1.23] | 0.451 |
|  | Atrial appendage closure | ● | 23 | 131 (2.6%) | 8 (4.5%) | 0.148 | 176 [0.85; 3.64] | 0.131 |
|  | Leukocytes first postop day (%) | ● | 19 | 9.9 [8.1;12.2] | 10.1 [8.3;13.3] | 0.082 | 1.01 [1.00; 1.02] | 0.054 |
|  | Leukocytes second postop day (%) | ● | 126 | 9.7 [8.1;11.7] | 10.7 [8.6;13.0] | <0.001 | 1.03 [1.01; 1.04] | 0.001 |
|  | Hb first postop day (g/dl) | ● | 19 | 10.4 [9.5;11.2] | 10.2 [9.6;10.9] | 0.261 | 0.96 [0.87; 1.05] | 0.348 |
|  | Hb second postop day (g/dl) | ● | 126 | 9.6 [8.8;10.5] | 9.5 [8.8;10.3] | 0.266 | 0.98 [0.91; 1.07] | 0.695 |
|  | Platelets first postop day (10^9^/L) | ● | 20 | 175 [144;212] | 176 [146;219] | 0.369 | 1.00 [1.00; 1.00] | 0.501 |
|  | Platelets second postop day (10^9^/L) | ● | 131 | 155 [127;189] | 155 [127;189] | 0.493 | 1.00 [1.00; 1.00] | 0.509 |
|  | INR first postop day | ● | 25 | 1.2 [1.2;1.3] | 1.3 [1.2;1.3] | 0.458 | 0.89 [0.35; 2.25] | 0.798 |
|  | INR second postop day | ● | 157 | 1.2 [1.1;1.3] | 1.2 [1.2;1.3] | 0.038 | 0.98 [0.84; 1.14] | 0.759 |
|  | CRP first postop day >3 mg/L ^a^ | ● | 117 | 4488 (91.0%) | 157 (90.8%) | 1.000 | 0.97 [0.57; 1.64] | 0.905 |
|  | CRP second postop day >3 mg/L^a^ | ● | 156 | 4881 (99.8%) | 171 (99.4%) | 0.362 | 0.42 [0.05; 3.25] | 0.406 |
|  | eGFR first postop day <60 ml/min ^b^ | ● | 22 | 1176 (23.4%) | 64 (36.4%) | <0.001 | 1.87 [1.37; 2.56] | <0.001 |
|  | eGFR second postop day <60 ml/min^b^ | ● | 126 | 1092 (22.2%) | 65 (38.0%) | <0.001 | 2.15 [1.57; 2.95] | <0.001 |
|  | Standard bone wax | ● | 1 | 425 (8.4%) | 16 (8.9%) | 0.918 | 1.07 [0.63; 1.80] | 0.810 |
|  | Water-soluble bone wax | ● | 0 | 834 (16.5%) | 21 (11.7%) | 0.108 | 0.67 [0.42; 1.10] | 0.090 |
|  | Antibiotic paste | ● | 0 | 168 (3.3%) | 6 (3.4%) | 1.000 | 1.01 [0.44; 2.30] | 0.988 |
|  | Fibrin sealant | ● | 0 | 168 (3.3%) | 14 (7.8%) | 0.003 | 2.46 [1.40; 4.34] | 0.002 |
| Legend: CI = confidence interval; CRP = C-reactive protein; DSWI = deep sternal wound infection; eGFR = estimated glomerular filtration rate – calculated by MDRD formula; INR = International Normalized Ratio; N.A. = logistic regression was not calculated, due to all cases of individual group were just in one category; OR = odds ratio. ^a^  categories were formed according to risk levels published in 2003 by American Heart Association & Centers for Disease Control and Prevention Scientific Statement [3]. ^b^ categories were formed according to NKF KDOQI GUIDELINES [4]. | | | | | | | | |

**Supplementary Table 3: Multivariable analysis of all included models**

| **Variable** | **BASELINE MODEL (M1)** | | **IMPROVED BASELINE MODEL (M2)** | | **EXTENDED MODEL (M3)** | |
| --- | --- | --- | --- | --- | --- | --- |
|  | **OR [95% CI]** | **P-value** | **OR [95% CI]** | **P-value** | **OR [95% CI]** | **P-value** |
| Female gender | 0.96 [0.65; 1.42] | 0.836 |  |  |  |  |
| BMI (kg/m^2^) >30 | 2.93 [2.12; 4.04] | <0.001 | 1.78 [1.04; 3.05] | 0.037 |  |  |
| Diabetes - On oral hypoglycaemic agents | 1.13 [0.78;1.63] | 0.527 |  |  |  |  |
| Diabetes - On insulin | 2.31 [1.56; 3.42] | <0.001 | 1.98 [1.34; 2.92] | 0.001 | 1.88[1.24; 2.57] | 0.003 |
| Poor glycaemic control | 0.98 [0.62; 1.57] | 0.947 |  |  |  |  |
| Chronic lung disease | 1.37 [0.91; 2.06] | 0.134 |  |  |  |  |
| Chronic dialysis | 0.68 [0.26; 1.77] | 0.428 |  |  |  |  |
| Congestive heart failure | 1.28 [0.91; 1.79] | 0.152 |  |  |  |  |
| Urgent surgical priority | 0.90 [0.65; 1.26] | 0.551 |  |  |  |  |
| BMI (kg/m^2^) |  |  | 1.11 [1.05; 1.17] | <0.001 | 1.16 [1.11; 1.20] | <0.001 |
| PAD |  |  | 1.92 [1.30; 2.83] | 0.001 | 2.19 [1.47; 3.26] | <0.001 |
| Preoperative hospital stay >24h |  |  | 1.47 [1.04; 2.09] | 0.031 | 1.77 [1.21; 2.57] | 0.003 |
| eGFR^1^ (ml/min) |  |  | 0.99 [0.98; 1.00] | 0.033 |  |  |
| LVEF (%) |  |  |  |  | 1.00 [0.97; 1.00] | 0.105 |
| eGFR^1^ (ml/min) <50 |  |  | 0.53 [0.30; 0.93] | 0.028 |  |  |
| Age (years) |  |  | 1.02 [0.99; 1.04] | 0.144 | 1.03 [1.00;1.05] | 0.020 |
| Acute kidney injury |  |  | 1.89 [1.25; 2.85] | 0.002 | 1.89 [1.24; 2.89] | 0.003 |
| LVEF (%) <50 |  |  | 1.41 [0.98; 2.05] | 0.068 |  |  |
| Porcelain aorta (by intraop. EAS) | 1.25 [0.89; 1.77] | 0.195 |  |  |  |  |
| Low cardiac output | 0.66 [0.31; 1.40] | 0.277 |  |  |  |  |
| Multiple blood transfusion (>2 RBCs) | 2.60 [1.76; 3.84] | <0.001 |  |  |  |  |
| Mediastinal re-exploration | 3.27 [1.93; 5.55] | <0.001 | 3.25 [1.93; 5.48] | <0.001 | 3.14 [1.82; 5.41] | <0.001 |
| Number of bypasses |  |  | 1.22 [0.98; 1.52] | 0.079 | 1.25 [0.99; 1.57] | 0.060 |
| Bilateral ITA |  |  | 5.22 [2.93; 9.31] | <0.001 | 4.81 [2.65; 8.73] | <0.001 |
| Prolonged (>48 h) invasive ventilation |  |  | 1.59 [0.95; 2.67] | 0.078 |  |  |
| Blood transfusion |  |  | 2.31 [1.60; 3.33] | <0.001 | 2.02 [1.38; 2.96] | <0.001 |
| eGFR^2^ first postop day <60 ml/min |  |  |  |  | 1.68 [1.45; 2.02] | 0.059 |
| Cardioversion |  |  |  |  | 1.62 [0.88; 3.01] | 0.124 |
| Fibrin sealant |  |  |  |  | 2.19 [1.10; 4.35] | 0.026 |
| Water-soluble bone wax |  |  |  |  | 0.66 [0.40; 1.10] | 0.109 |
| Postoperative delirium |  |  |  |  | 2.04 [1.32; 3.16] | 0.001 |
| Pleural effusion - intervention |  |  |  |  | 2.26 [1.53; 3.34] | <0.001 |
| LEGEND: BMI = Body Mass Index; CI = confidence interval; EAS = epiaortic ultrasonography scan; eGFR^1^ = estimated glomerular filtration rate – calculated by Cockcroft-Gault formula; eGFR^2^ = estimated glomerular filtration rate – calculated by MDRD formula; ITA = internal thoracic artery; LVEF = left ventricular ejection fraction; OR = odds ratio;PAD = peripheral arterial disease; RBCs = packed red blood cells. | | | | | | |

**REFERENCES**

1. Gatti G, Dell’Angela L, Barbati G, Benussi B, Forti G, Gabrielli M, et al. A predictive scoring system for deep sternal wound infection after bilateral internal thoracic artery grafting. Eur J Cardiothorac Surg. 2016;49:910–7.

2. Raja SG, Benedetto U. Scoring system to guide decision making for the use of bilateral internal mammary arteries: The BIMA score. Int J Surg. 2018;51:89–96.

3. Pearson TA, Mensah GA, Alexander RW, Anderson JL, Cannon RO, Criqui M, et al. Markers of Inflammation and Cardiovascular Disease: Application to Clinical and Public Health Practice: A Statement for Healthcare Professionals From the Centers for Disease Control and Prevention and the American Heart Association. Circulation. 2003;107:499–511.

4. National Kidney Foundation. K/DOQI clinical practice guidelines for chronic kidney disease: evaluation, classification, and stratification. Am J Kidney Dis. 2002;39:S1-266.
